# Real-world analysis of healthcare resource utilization by patients with X-linked myotubular myopathy (XLMTM) in the United States

**DOI:** 10.1186/s13023-023-02733-2

**Published:** 2023-06-06

**Authors:** Robert J. Graham, Basil T. Darras, Tmirah Haselkorn, Dan Fisher, Casie A. Genetti, Weston Miller, Alan H. Beggs

**Affiliations:** 1 Division of Critical Care Medicine, Boston Children’s Hospital, Harvard Medical School, Boston, MA USA; 2 Department of Neurology, Neuromuscular Program, Boston Children’s Hospital, Harvard Medical School, Boston, MA USA; 3 Astellas Gene Therapies, San Francisco, CA USA; 4 IPM.Ai, Florham Park, NJ USA; 5 Division of Genetics and Genomics, The Manton Center for Orphan Disease Research, Boston Children’s Hospital, Harvard Medical School, 3 Blackfan Circle - BCH3150, Boston, MA 02115 USA; 6 Formerly of Astellas Gene Therapies, San Francisco, CA USA

**Keywords:** X-linked myotubular myopathy, Congenital myopathy, Insurance claims analysis

## Abstract

**Background:**

X-linked myotubular myopathy (XLMTM) is a rare, life-threatening congenital myopathy with multisystem involvement, often requiring invasive ventilator support, gastrostomy tube feeding, and wheelchair use. Understanding healthcare resource utilization in patients with XLMTM is important for development of targeted therapies but data are limited.

**Methods:**

We analyzed individual medical codes as governed by Healthcare Common Procedure Coding System, Current Procedural Terminology, and International Classification of Diseases, 10th Revision (ICD-10) for a defined cohort of XLMTM patients within a US medical claims database. Using third-party tokenization software, we defined a cohort of XLMTM patient tokens from a de-identified dataset in a research registry of diagnostically confirmed XLMTM patients and de-identified data from a genetic testing company. After approval of an ICD-10 diagnosis code for XLMTM (G71.220) in October 2020, we identified additional patients.

**Results:**

A total of 192 males with a diagnosis of XLMTM were included: 80 patient tokens and 112 patients with the new ICD-10 code. From 2016 to 2020, the annual number of patients with claims increased from 120 to 154 and the average number of claims per patient per year increased from 93 to 134. Of 146 patients coded with hospitalization claims, 80 patients (55%) were first hospitalized between 0 and 4 years of age. Across all patients, 31% were hospitalized 1–2 times, 32% 3–9 times, and 14% ≥ 10 times. Patients received care from multiple specialty practices: pulmonology (53%), pediatrics (47%), neurology (34%), and critical care medicine (31%). The most common conditions and procedures related to XLMTM were respiratory events (82%), ventilation management (82%), feeding difficulties (81%), feeding support (72%), gastrostomy (69%), and tracheostomy (64%). Nearly all patients with respiratory events had chronic respiratory claims (96%). The most frequent diagnostic codes were those investigating hepatobiliary abnormalities.

**Conclusions:**

This innovative medical claims analysis shows substantial healthcare resource use in XLMTM patients that increased over the last 5 years. Most patients required respiratory and feeding support and experienced multiple hospitalizations throughout childhood and beyond for those that survived. This pattern delineation will inform outcome assessments with the emergence of novel therapies and supportive care measures.

**Supplementary Information:**

The online version contains supplementary material available at 10.1186/s13023-023-02733-2.

## Introduction

In X-linked myotubular myopathy (XLMTM), mutations of the *MTM1* gene that encodes myotubularin protein lead to myotubularin deficiency and a rare, life-threatening congenital myopathy [1] with estimated incidence of 1 in 40,000–50,000 newborn males [2]. Myotubularin is required for normal development, maturation, and maintenance of skeletal muscle cells; thus, deficiency affects skeletal muscles throughout the body [1, 3, 4]. Historically, approximately half of affected male infants died before 18 months of age, most often related to respiratory failure [5–7]. These children rely on intensive medical intervention for survival [5–7].

Natural history studies have demonstrated multisystem involvement in XLMTM and cumulative morbidities that contribute to extensive disease burden. These include respiratory failure and resulting need for invasive ventilator support, feeding and swallowing impairments and associated aspiration risks leading to need for gastrostomy tube feeding, inability to ambulate independently requiring wheelchair use, absent or delayed attainment of major motor milestones, scoliosis and other musculoskeletal morbidities, and hepatobiliary disease and hepatic vascular abnormalities, such as peliosis [5, 6, 8–10].

Presently, there are no approved treatments that alter the disease pathology in XLMTM and no established consensus on disease management. Multidisciplinary supportive care for the aforementioned multisystemic manifestations of chronic, profound muscle weakness is required to prolong life and support mobility [5–7]. Multisystem disease involvement means that management and care of XLMTM is broad and complex and diagnosis, established or confirmed by genetic testing, can involve numerous diagnostic procedures and extensive healthcare resource utilization. Direct medical costs and health care resource utilization for children with XLMTM were recently estimated at $897,978 per patient in the first year of life and nearly $1.5 million total for patients who survive the first 4 years [11]**.**

Although significant numbers of carrier females experience variable degrees of disability [12], we limited this analysis to males with XLMTM as they represent the large majority of affected individuals and experience complete penetrance of pathogenic mutations in the hemizygous state [2]. To further refine our understanding of the disease burden imposed by XLMTM we utilized a novel approach to claims analysis involving a comprehensive insurance claims database and artificial intelligence capabilities to quantify the disease burden of XLMTM, including multidisciplinary disease management; multiple comorbid conditions; and the need for hospitalization, invasive ventilation, and feeding support. In addition, the release of an International Classification of Diseases, 10th Revision (ICD-10) code for XLMTM in October 2020 allowed for more precise identification and assessment of these patients.

## Methods

### Data collection

We set out to identify a known cohort of male XLMTM patients within IPM.ai’s (Florham Park, NJ, USA) licensed patient-level data, consisting of US medical and pharmacy claims for more than 300 million unique patients across commercial and Center for Medicare and Medicaid services (CMS) payors. Typically, Boolean logic can be applied to medical coding (diagnosis, prescription, and procedure codes) using the ICD-10 code to arrive at the closest approximation of the confirmed patient cohort. However, until the approval of a specific ICD-10 code for XLMTM in October 2020, XLMTM was most commonly grouped with other myopathies under the G71.2 (congenital myopathies) ICD-10 code, which is not specific to XLMTM. Therefore, we needed to leverage an external data source to more accurately define an XLMTM patient cohort.

A de-identified dataset of XLMTM patients from an existing research registry of molecularly confirmed XLMTM patients at Boston Children’s Hospital was utilized to define a derivation set of patients. In order to maintain patient privacy, the statistical authors (IPM.ai, Florham Park, NJ, USA) engaged a third-party application (Datavant, San Francisco, CA, USA) to complete HIPAA-compliant patient tokenization (i.e., the process of replacing sensitive data with non-sensitive placeholders called “tokens”). The datasets in this publication were linked using privacy-preserving record linkage provided by Datavant [13]. Datavant’s solution uses personally identifiable information to create encrypted tokens, which are de-identified, unique and irreversible patient keys. Tokens are used to match individual records across different data sets without ever exposing the personally identifiable information of the patient to whom each record belongs. Thus, protected health information was not shared between the patient registry and the IPM.ai claims dataset.

The dataset consisted of 113 de-identified patient tokens, of which 76 matched the claims dataset. By engaging a genetic testing company (Invitae, San Francisco, CA, USA), Astellas Gene Therapies also purchased data on XLMTM patients confirmed through diagnostic testing. Through the same third-party tokenization software, IPM.ai incrementally identified four additional XLMTM patients, increasing the study population to 80 patient tokens matched in the claims dataset.

Finally, since the approval of ICD-10 diagnosis code G71.220 for XLMTM in October 2020, 135 male patients have been coded with G71.220, 23 of whom were previously identified through patient registry or genetic testing tokens, yielding a net increase of 112 patients for a total analysis population of 192 unique patients.

### Medical code grouping

The research team analyzed individual medical codes as governed by Healthcare Common Procedure Coding System (HCPCS), Current Procedural Terminology (CPT), and the ICD-10. While each code carries specific information on care delivered to the patient, it is often the case that a diagnosis may have multiple sub-codes. Reporting the frequency with which a cohort of patients presents with a diagnosis of interest requires grouping like codes together, so as to track the overall occurrence of the disease. For example, a patient with scoliosis may have in their history a code for “Scoliosis, unspecified” (M41.9), as well as “Other forms of scoliosis, lumbar region” (M41.86). If each code were reported independently, one could not ascertain whether the same patient appeared with one or both codes. To ensure the number of patients with each comorbidity or procedure was as accurate and comprehensive as possible, a cross-functional team of physicians reviewed and aligned on the individual medical codes that were deemed appropriate to group together.

### Physician identifier remapping

Health care providers are most commonly identified in data using their National Provider Identifier (NPI), a unique 10-digit identification number containing their profile detail, including name, address, and self-reported specialty. It is not unusual for provider specialty data to be inaccurate, whether due to further sub-specialization or original mis-capture of the data. The research team conducted a process of reconciling NPI details by constructing a web-scraping tool and overriding provider specialty using the most recent data captured on their practice biography. All data contained in the analysis—namely, care utilization by specialty—are based on this revised NPI information.

### Approval for use of Boston Children’s Hospital Registry

One of the authors (Dr. Beggs) directs a research study with an umbrella protocol (Protocol number 03-08-128R) approved by the Boston Children’s Hospital institutional review board (IRB) entitled “Molecular Analysis of Neuromuscular Disease.” Under the terms of this protocol, the informed consent form specifies that de-identified data and samples may be shared with researchers outside Boston Children’s Hospital for related studies of neuromuscular disease led by other qualified investigators, academic institutions, and businesses. To permit use of the tokenization software behind the Boston Children’s Hospital secure firewall, the protocol and software package underwent security review by the Boston Children’s Hospital Clinical Research Informatics Team, which approved its use for the described purpose of deidentifying the Beggs Study cohort of XLMTM patients. That review was accepted by the (IRB), which subsequently approved an amendment to the IRB protocol allowing tokenization and sharing of tokens with IPM.ai and Astellas Gene Therapies (formerly, Audentes Therapeutics). All storage, manipulation, and processing of patient identifiers to generate tokens was performed on secure encrypted computing devices protected by the Boston Children’s Hospital institutional firewall.

### Statistical analysis

In every case where there are fewer than or equal to three patients, HIPAA suppression logic was applied to remove the risk of reidentification. All patients who met this criterion were subsequently noted with the value of “≤ 3” and no efforts were made to reidentify or reverse engineer any volumes of patients. The absence of a code did not necessarily mean that the event did not occur, due to conventional coverage gaps that occur in open claims datasets. Unless otherwise suppressed for compliance purposes, all cohort *n* sizes are indicated. Where *n* sizes are 30 or fewer patients, analyses should be interpreted as directional. All analyses were performed using an open universe of claims dataset, with the queries written in SQL code. Descriptive statistics (frequencies and proportions) were generated for categorical variables.

## Results

### Patients and providers

**Fig. 1:**
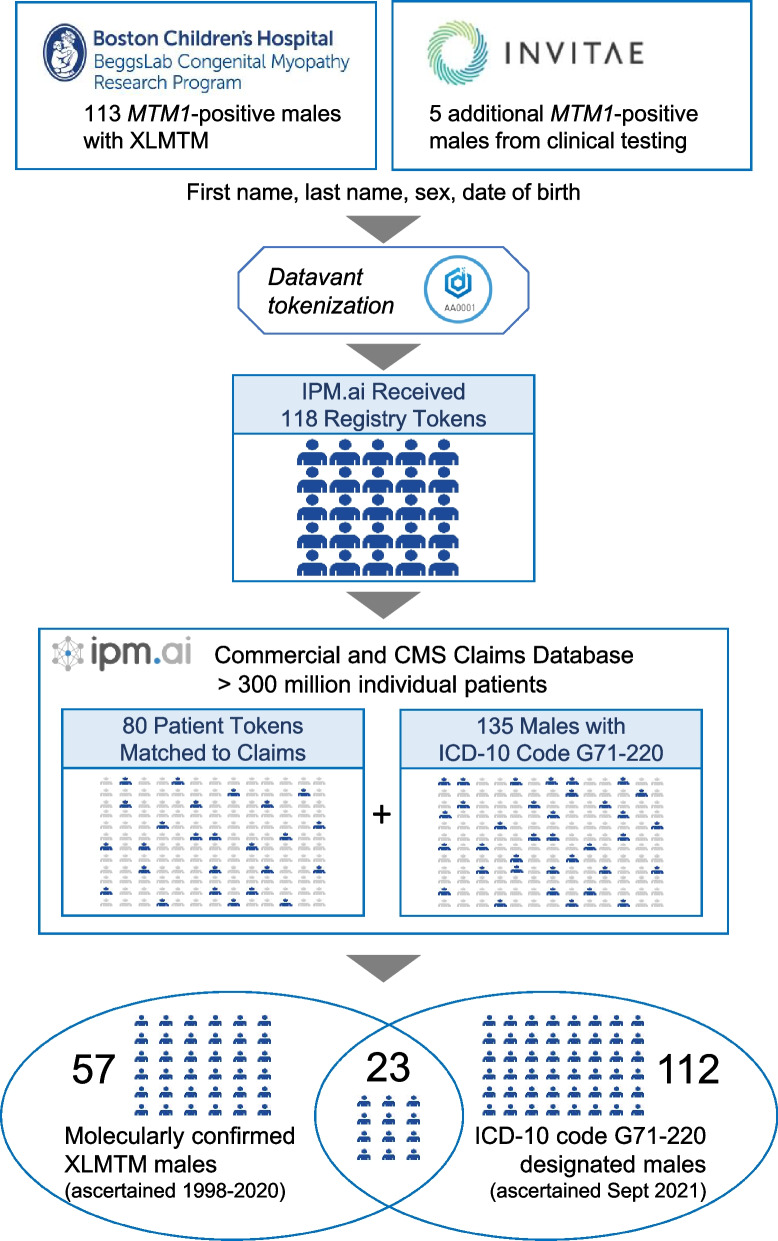
Flow diagram illustrating methods of ascertainment of study cohort subpopulations and the flow of information through the tokenization and data extraction process

**Fig. 2:**
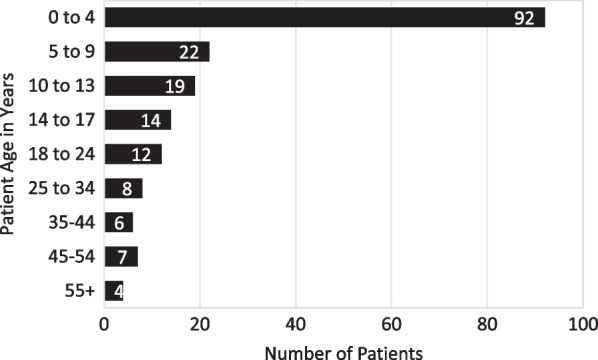
Age at first known congenital myopathy diagnosis in XLMTM patients (N = 184). Actual first congenital myopathy claim could be at an earlier age depending on the amount of patient history in the dataset. Eight patients did not have a congenital myopathy diagnosis

Our analysis included 192 males with a diagnosis of XLMTM, including 80 patient tokens and 112 patients classified with the new XLMTM ICD-10 code (Fig. 1). Fifty percent of patients were 0 to 4 years of age when they received their first congenital myopathy diagnosis (Fig. 2). At the time of the last reported claim for each patient, 121 patients (63%) were < 18 years old, 31 patients (16%) were 18–24 years old, 20 patients (10%) were 25–34 years old, and 20 patients (10%) were ≥ 35 years old.

XLMTM patients with a congenital myopathy diagnosis received care from a variety of specialty practices (Table 1). Approximately half were seen in pulmonology (53%) and pediatric practices (47%) and about one-third were seen by neurology (34%) and critical care medicine (31%). Regionally, most of the health care providers caring for XLMTM patients were located in the eastern half of the US and along the west coast (Additional file 1: Fig. S1).

**Table 1:** Specialists seen by XLMTM patients with a congenital myopathy diagnosis (N = 124)

Specialty*	No. of patients	Percent of patients
Pulmonology	101	53%
Pediatrics	91	47%
Neurology	66	34%
Critical Care Medicine	60	31%
Emergency Medicine	46	24%
Otolaryngology	37	19%
Gastroenterology	37	19%

*“Pediatrics” represents general pediatricians not specialized in any particular organ system. Other categories can be assumed to represent specialists in either pediatric or adult medicine as appropriate for the age distribution of the patient population

**Fig. 3:**
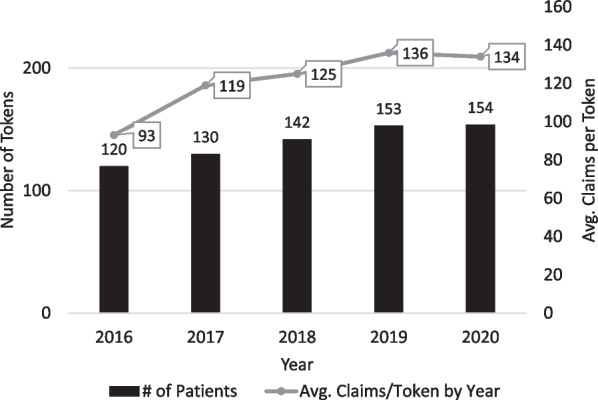
Claims activity by year

During the study period from 2016 to 2020, the number of patients with claims each year increased steadily from 120 to 154 and the average number of claims per patient per year increased from 93 to 134 (Fig. 3).

### Diagnostic and therapy utilization

The most common conditions and procedures related to XLMTM (Table 2) reflect high disease burden, including respiratory events (82%), ventilation management (82%), tracheostomy (64%), feeding difficulties (81%), feeding support (72%), gastrostomy (69%), scoliosis (42%), other myopathies (77%), wheelchair equipment (56%), and critical and emergency care (71%). Among patients who experienced respiratory events, most experienced acute respiratory distress or arrest (54%), nearly all experienced chronic respiratory events (96%), and others had medical coding that classified respiratory issues as acute and chronic (46%). Medical codes were classified as acute when explicitly stated as “acute” in the ICD-9 or ICD-10 code; medical codes were classified as chronic when explicitly stated as “chronic” in ICD-9 or ICD-10 coding or could not be ascertained from the code description as acute or chronic; any codes described as “acute and chronic” were labeled as such.

**Table 2:** Common conditions and procedures/services related to XLMTM in the overall XLMTM population with a diagnosis of congenital myopathy

	Percent of patients (N = 192)
*Conditions*	
Respiratory distress, failure, arrest	82%
Acute	54%
Chronic	96%
Acute and chronic	46%
Feeding difficulties and related problems	81%
Gastrostomy*	69%
Tracheostomy*	64%
Ventilator use or dependence	61%
Other myopathies	77%
Scoliosis	42%
Liver disorders	33%
Wheelchair dependence	21%
*Procedures/services*	
Ventilation management or equipment	82%
Feeding support	72%
Critical or emergency care	71%
Tracheostomy (procedure or supplies)*	62%
Wheelchair equipment	56%
Gastrostomy (procedure)*	52%
Hospital/Inpatient care	46%
Occupational Therapy or Phyiscal Therapy	49%
Home care	35%

^*^Gastrostomy and tracheostomy coding are each based on the presence of ICD-10 diagnosis coding. When available in diagnosis coding, patients are counted under “Conditions.” When procedures or durable equipment associated with gastrostomy and tracheostomy are available in CPT coding or ICD-10 procedures, patients are counted under “Procedures/Services”

**Fig. 4:**
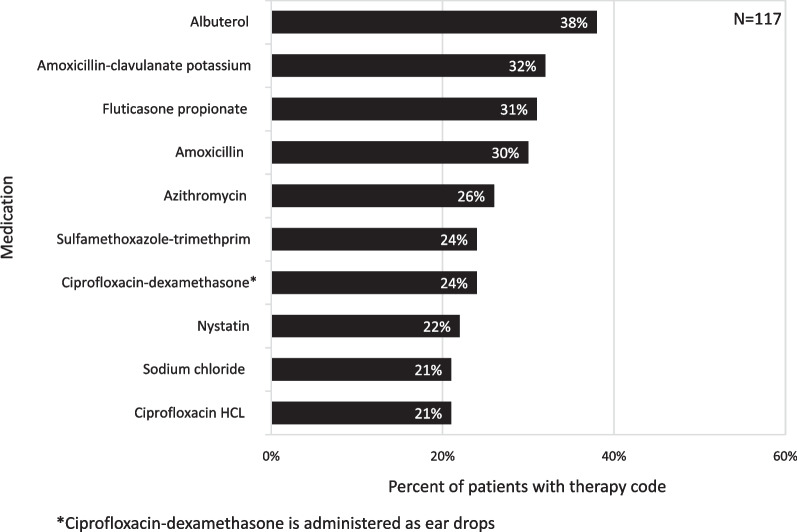
Common medications

The most commonly prescribed medications (Fig. 4) were those often used to treat respiratory illness and infection, including albuterol (38%), amoxicillin-clavulanate (32%), fluticasone propionate (31%), and amoxicillin (30%). The most frequently used diagnosis and procedure codes (Table 3), however, were for investigation of various cholestatic and liver abnormalities, including, gamma-glutamyl transferase [25]; abnormal serum enzyme; other or unspecified [21]; jaundice [17]; elevation of aspartate aminotransferase (AST), alanine aminotransferase (ALT), or lactate dehydrogenase (LDH) [12]; obstruction of gallbladder or bile duct [11]; and cholelithiasis [10]. Abnormal living function/imaging was present in 16 (8%) patients, disorders of bilirubin metabolism in 9 (5%) patients, and the combination of abnormal serum enzyme levels and disorders of bilirubin metabolism in 27 (14%) patients.

**Table 3:** Frequency of applicable diagnosis and procedure codes searched across 192 patients

Diagnosis or Procedure Description	Code(s)	Frequency
Abnormal serum enzyme, other or unspecified	ICD9: 790.5 & ICD10: R74.X	21
Alpha-1-antitrypsin deficiency	ICD9: 273.4 & ICD10:E88.01	≤ 3
Ascites	ICD9: 789.59 & ICD10: R18.8	6
Biliary atresia, congenital	ICD9: 751.61 & ICD10: Q44.2	≤ 3
Cholangitis	ICD9: 576.1 & ICD10: K83.0	0
Cholecystitis	ICD9: 575.x & ICD10: K81.x	≤ 3
Cholelithiasis	ICD9: 574.x & ICD10: K80.x	10
Cholelithiasis w/ obstruction	ICD9: 574.x & ICD10: K80.x	0
Cholesterolosis	ICD9: 576.6 & ICD10: K82.4	0
Disorders of bilirubin metabolism	ICD9: 277.4 & ICD10: E80.x	9
Elevation of AST, ALT, or LDH	ICD9: 790.4 & ICD10: R74.0	12
Endoscopic retrograde cholangiopancreatography (ERCP)	CPT: 43260-43278 & CPT: 0397T	0
Galactosemia	ICD9: 271.1 & ICD10: E74.21	0
Gamma-glutamyl transferase	CPT: 82977	25
Hepatitis, infectious	ICD9: 070.x, 091.62, 130.5, 573.x, & ICD10: B00,81, B15.x, B16.x, B17.x, B18.x, B19.x, B25.1, B26.81, B58.1, B94.2, O98.x, V02.x	≤ 3
Hepatitis, infectious, neonatal	ICD10: P35.3	0
Hepatitis, non-infectious	ICD9: 571.x, 573.3 & ICD10: K73.x, K75.x	6
Hepatomegaly	ICD9: 789.1 & ICD10: R16.x	6
Jaundice	ICD9: 782.4 & ICD10: R17	17
Jaundice, neonatal	ICD9: 774.x & ICD10: P58.x, P59.x	9
Laparoscopic cholecystectomy	CPT: 47562-47564	≤ 3
Lithotripsy	CPT: 50590	≤ 3
Magnetic resonance cholangiopancreatography (MRCP)	CPT: S8037	0
Obstruction of gallbladder or bile duct	ICD9: 575.2, 576.2 & ICD10: K82.0, K83.1	11
Other congenital malformation of bile ducts, liver or gallbladder	ICD9: 751.x & ICD10: Q44.x	≤ 3
Other disease of biliary tract	ICD9: 576.x & ICD10: K83.x, Q44.4, Q44.5	7
Other disease of gallbladder	ICD9: 575.X, 576.3 & ICD10: K82.X	5
Other liver diseases	ICD9: 571.x, 573.x, 751.x, & ICD10: K76.x, Q44., Q44.7	0
Pruritus	ICD9: 698.x & ICD10: L29.x	6
Toxic liver disease	ICD10: K71.x	0

AST: aspartate aminotransferase; ALT: alanine aminotransferase; CPT: Current Procedural Terminology; ICD: International Classification of Disease; LDH: lactate dehydrogenase

### Inpatient claims

The total number of observed annual inpatient claims increased from 5,914 in 2016 to 11,513 in 2020 and the average number of inpatient claims per patient annually increased from 57 to 92 over the same time period (Table 4).

**Table 4:** Average number of annual inpatient claims across all patients in aggregate

Year	Total number of inpatient claims	Unique XLMTM patients in dataset	Average number of annual inpatient claims per XLMTM patient
2016	5914	103	57
2017	7417	102	73
2018	9580	108	89
2019	11,084	128	87
2020	11,513	125	92

Data are based on claims with an inpatient hospital place of service designation

**Fig. 5:**
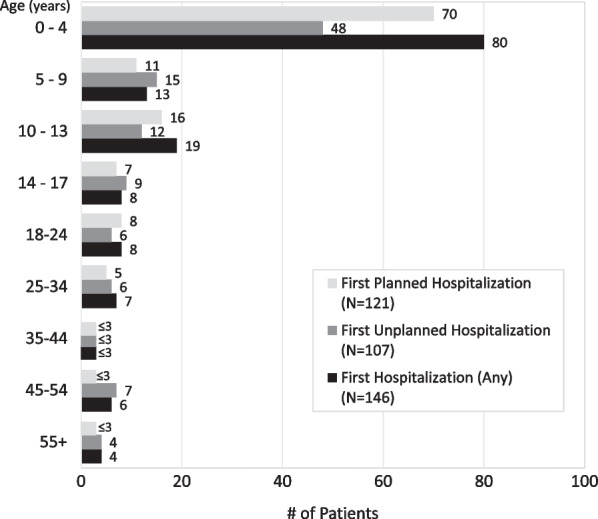
Age at first hospitalization (any, unplanned, and planned)

**Fig. 6:**
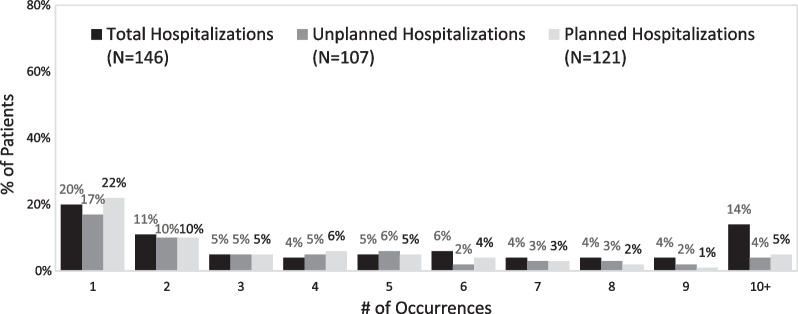
Number of hospitalizations (any, unplanned, and planned)

Hospitalizations, excluding normal birth deliveries, were analyzed in aggregate (n = 146), then further segmented based on unplanned hospitalizations (n = 107) and planned hospitalizations (n = 121) (coding provided in Additional file 1: Table S1). Approximately half of patients (55%, n = 80) experienced their first hospitalization between 0 and 4 years of age: across all age groups, 76% experienced any hospitalization, 55% for unplanned and 63% for planned hospitalizations Fig. 5). The greatest proportion of patients experienced 1 to 2 hospitalizations (31% any, 27% unplanned and 32% planned) (Fig. 6).

## Discussion

This study utilized a novel claims data analysis involving a comprehensive insurance claims database and artificial intelligence capabilities to quantify the disease burden of XLMTM. Results document the substantial disease burden of XLMTM, including extensive health care resources needed to care for these patients, specifically respiratory and ventilator support, tracheostomy, gastrostomy tube feeding, wheelchair use, critical and emergency care, medications for respiratory illness and infections, and hospitalizations. Caring for these patients required multispecialty involvement (pulmonology, pediatrics, neurology and critical care medicine) and high rates of multiple hospitalizations, surgical procedures, and use of assistive devices. Notably, the average number of inpatient claims per patient aged 0 to 4 and 5 to 12 were 23.2 and 23.8, respectively. Among age- and gender-matched controls in the IPM.ai claims database representing the general population, the average inpatient claims per year for patients 0 to 4 years and 5 to 12 years of age were 0.3 for both groups (Table 5). Interestingly, the number of claims increased between 2016 and 2020. It is not apparent from the data what might account for this, but the shift from ICD-9 to ICD-10 in October 2015 may have increased granularity in the later years and the new XLMTM-specific ICD-10 code and increased genetic testing in general might have contributed to improved data capture.

**Table 5:** Average inpatient claims per year for patients with XLMTM and the general population

Age Group	Average claims per year per XLMTM patient	Average claims per year per general population*
0–4 years old (N = 86)	23.2	0.3
5–12 years old (N = 85)	23.8	0.3
13–17 years old (N = 45)	14.1	0.2
18+ years old (N = 60)	18.0	0.4

*Source: IPM.ai claims dataset, consisting of > 300 million US patient lives

These findings are largely consistent with the high disease burden associated with need for mechanical ventilation, gastrostomy tube feeding, and wheelchair requirement reflected in the limited natural history studies of XLMTM [5–9], including the RECENSUS retrospective chart review of 112 males with XLMTM. This is likely due, in part, to a high degree of patient overlap between the RECENSUS study cohort [5, 6] and this claims analysis data set (76 of the patient tokens in our study were also RECENSUS patients). However, the RECENSUS study focused on disease manifestations and recorded medical management and was limited by incomplete availability of data, while our all-claims analysis represents a more complete overview of all aspects of the patients’ medical care. For example, in the RECENSUS study, codes for diagnosis of hepatobiliary disease were among the most common ICD-10 codes utilized despite hepatobiliary disease not being a primary manifestation of XLMTM. Use of a certain ICD-10 code, however, does not mean the patient has the disease of interest. Physicians often refer XLMTM patients to gastroenterology for liver evaluation, using a code for liver abnormality despite the absence of overt liver disease. This finding of high use of hepatobiliary ICD-10 codes highlights an area requiring further study in light of recent reports of hepatobiliary abnormalities as an under-appreciated aspect of XLMTM, including cholestasis [14, 15], hypertransaminemia and hyperbilirubinemia with hepatopathy and cholestasis [16, 17].

Our claims analysis expands on the findings from an economic analysis by Sacks et al. showing very high medical costs and intense healthcare resource utilization associated with caring for patients with XLMTM [11]. First hospitalization between 0 and 4 years of age for 80% of patients in our study supports Sack et al.’s estimate of mean monthly per patient direct medical costs being highest in the first year of life ($74,831), with declining costs over the second, third, and fourth years of life ($23,207, $13,044, and $9,440, respectively) [11]. In addition, inpatient admissions ($69,025) accounted for the majority of the mean monthly per patient direct medical costs [11]. Both studies identified the major burden of ventilator support and ventilation management. Moreover, our study quantified the extensive use of tracheostomy, gastrostomy tube feeding, wheelchairs, occupational and physical therapy, and home care services. The Sacks et al. analysis estimated the mean monthly per patient prescription medication cost at $540 [11] and we have shown that the most common medications prescribed for these patients are medications for respiratory illnesses and infections.

The use of deidentified tokens for record matching across research consortia and between identified research databases and anonymized public databases has been growing [18], and a recent study reported 99% precision for matching among 20,002 record pairs when first name, last name, gender, and date of birth were tokenized [19]. However, as this analysis employed a novel use of artificial intelligence capabilities on both commercial and CMS payor databases, namely Medicaid claims, it has inherent limitations. Capture of open claims data relies on billing codes that may not accurately reflect the care delivered or the clinical reasoning for ordering a given test or procedure. In addition, the existing challenges of using nonspecific congenital myopathy ICD-10 codes in research on XLMTM were further complicated by the release of the new XLMTM-specific diagnosis code during the study time frame. Given the newness of this code, it will be important to validate how rigorously it is being applied, and whether care providers are limiting its use to patients with genetically confirmed diagnoses. Finally, the need to group like codes together into a code set for reporting purposes of some data limited the granularity of our analysis. Similarly, our age-group coding was structured to group events within the patients first 4 years of life in order to avoid reporting distinct patient counts fewer than four patients. However, this approach precludes looking specifically at coding for events within the first year of life, which could be useful in the setting of XLMTM research. We also saw an unexpectedly high number (n = 20) of XLMTM patients older than 35 years, 17 of whom were identified of the basis of ICD-10 code, which raises the question of whether some older males with congenital myopathy on muscle biopsy are being coded as XLMTM without the benefit of genetic testing. Thus, although at least three patients with molecular confirmation of *MTM1* mutations were over the age of 35, analysis of age at diagnosis and first hospitalization (Figs. 2 and 5) should be considered in light of the possibility that some of the other 17 might reflect overuse of the XLMTM ICD-10 diagnostic code.

Due to the nature of open source databases, our findings likely underestimate the XLMTM disease burden. While the IPM.ai dataset (and others like it) represents CMS and privately insured patients, there are coverage gaps when using open source data. In particular, the available in-hospital data often include only admission and discharge data and the events happening within the hospital stay are not captured. Nonetheless, our analysis is more broadly representative of the general US population than the more idiosyncratic RECENSUS dataset, which was based on a research program from a specific patient registry.

While currently there are no disease-modifying therapies approved to treat XLMTM, several novel therapeutic strategies have shown promise in pre-clinical disease models, including tamoxifen [20], PIK3C2B inhibition [21], dynamin 2 reduction [22], and intramuscular injections of either adeno-associated virus (AAV)-shRNA or AAV-mediated gene replacement therapy (resamirigene bilparvovec) [23–26]. Our improved understanding of the unmet medical burden reflected in these claims data will enable rigorous and appropriate risk–benefit considerations as these and other therapies are developed and become available.

## Conclusions

To our knowledge, this is the first analysis to combine a comprehensive claims database with artificial intelligence capabilities to quantify the extensive disease burden and health care resources needed to provide patients with XLMTM the care they rely on for survival. These data will be valuable for assessing healthcare resource utilization for future disease-modifying therapies relative to the current supportive care.

## Supplementary Information

**Additional file 1. Figure S1.** Regional heat map of healthcare provider locations for all 192 patients based on the NPI Registry Practice by 5-digit zip code. **Table S1.** Codes for planned vs unplanned hospitalization.

## Data Availability

The datasets used and/or analyzed during the current study are available based on Astellas criteria on data sharing availability at https://clinicalstudydatarequest.com/Study-Sponsors/Study-Sponsors-Astellas.aspx.

## Abbreviations

AAV: Adeno-associated virus
CMS: Center for Medicare and Medicaid services
CPT: Current Procedural Terminology
HCPCS: Healthcare Common Procedure Coding System
HIPAA: Health Insurance Portability and Accountability Act
ICD-10: International Classification of Diseases, 10th revision
IRB: Institutional review board
NPI: National Provider Identifier
XLMTM: X-linked myotubular myopathy

## References

[1] Laporte J, Biancalana V, Tanner SM (2000). *MTM1* mutations in X-linked myotubular myopathy. Hum Mutat.

[2] Vandersmissen I, Biancalana V, Servais L (2018). An integrated modelling methodology for estimating the prevalence of centronuclear myopathy. Neuromuscul Disord.

[3] Lawlor MW, Beggs AH, Buj-Bello A (2016). Skeletal muscle pathology in X-linked myotubular myopathy: review with cross-species comparisons. J Neuropathol Exp Neurol.

[4] Raess MA, Friant S, Cowling BS (2017). WANTED—dead or alive: Myotubularins, a large disease-associated protein family. Adv Biol Regul.

[5] Beggs AH, Byrne BJ, De Chastonay S (2018). A multicenter, retrospective medical record review of X-linked myotubular myopathy: The RECENSUS study. Muscle Nerve.

[6] Graham RJ, Muntoni F, Hughes I (2020). Mortality and respiratory support in X-linked myotubular myopathy: a RECENSUS retrospective analysis. Arch Dis Child.

[7] McEntagart M, Parsons G, Buj-Bello A (2002). Genotype-phenotype correlations in X-linked myotubular myopathy. Neuromuscul Disord.

[8] Amburgey K, Tsuchiya E, de Chastonay S (2017). A natural history study of X-linked myotubular myopathy. Neurology.

[9] Annoussamy M, Lilien C, Gidaro T (2019). X-linked myotubular myopathy: A prospective international natural history study. Neurology.

[10] Herman GE, Finegold M, Zhao W (1999). Medical complications in long-term survivors with X-linked myotubular myopathy. J Pediatr.

[11] Sacks NC, Healey BE, Cyr PL (2021). Costs and health resource use in patients with X-linked myotubular myopathy: insights from US commercial claims. J Manag Care Spec Pharm.

[12] Cocanougher BT, Flynn L, Yun P (2019). Adult MTM1-related myopathy carriers: classification based on deep phenotyping. Neurology.

[13] Datavant. Overview of Datavant's de-identification and linking technology for structured data. https://datavant.com/wp-content/uploads/dlm_uploads/2018/09/WhitePaper_-De-Identifying-and-Linking-Structured-Data.pdf. Accessed March 14, 2022.

[14] Molera C, Sarishvili T, Nascimento A (2021). Intrahepatic cholestasis is a clinically significant feature associated with natural history of X-linked myotubular myopathy (XLMTM): a case series and biopsy report. J Neuromuscul Dis.

[15] D'Amico A, Longo A, Fattori F (2021). Hepatobiliary disease in XLMTM: a common comorbidity with potential impact on treatment strategies. Orphanet J Rare Dis.

[16] Gangfuss A, Schmitt D, Roos A (2021). Diagnosing X-linked myotubular myopathy—a German 20-year follow up experience. J Neuromuscul Dis.

[17] Neese JM, Yum S, Matesanz S (2021). Intracranial hemorrhage secondary to vitamin K deficiency in X-linked myotubular myopathy. Neuromuscul Disord.

[18] Kiernan D, Carton T, Toh S (2022). Establishing a framework for privacy-preserving record linkage among electronic health record and administrative claims databases within PCORnet((R)), the National Patient-Centered Clinical Research Network. BMC Res Notes.

[19] Bernstam EV, Applegate RJ, Yu A (2022). Real-world matching performance of deidentified record-linking tokens. Appl Clin Inform.

[20] Maani N, Sabha N, Rezai K (2018). Tamoxifen therapy in a murine model of myotubular myopathy. Nat Commun.

[21] Sabha N, Volpatti JR, Gonorazky H (2016). PIK3C2B inhibition improves function and prolongs survival in myotubular myopathy animal models. J Clin Invest.

[22] Cowling BS, Chevremont T, Prokic I (2014). Reducing dynamin 2 expression rescues X-linked centronuclear myopathy. J Clin Invest.

[23] Buj-Bello A, Fougerousse F, Schwab Y (2008). AAV-mediated intramuscular delivery of myotubularin corrects the myotubular myopathy phenotype in targeted murine muscle and suggests a function in plasma membrane homeostasis. Hum Mol Genet.

[24] Childers MK, Joubert R, Poulard K (2014). Gene therapy prolongs survival and restores function in murine and canine models of myotubular myopathy. Sci Transl Med.

[25] Elverman M, Goddard MA, Mack D (2017). Long-term effects of systemic gene therapy in a canine model of myotubular myopathy. Muscle Nerve.

[26] Mack DL, Poulard K, Goddard MA (2017). Systemic AAV8-mediated gene therapy drives whole-body correction of myotubular myopathy in dogs. Mol Ther.

