# Supplementary material for: Real-world analysis of healthcare resource utilization by patients with X-linked myotubular myopathy (XLMTM) in the United States
Source: Orphanet J Rare Dis. 2023 Jun 6;18:138. doi: 10.1186/s13023-023-02733-2 (PMC10242920; doi:10.1186/s13023-023-02733-2)
Supplement: Supplementary file 1 — Additional file 1. Figure S1. Regional heat map of healthcare provider locations for all 192 patients based on the NPI Registry Practice by 5-digit zip code. Table S1. Codes for planned vs unplanned hospitalization. [file 13023_2023_2733_MOESM1_ESM.docx]

Supplementary Material

**Figure S1. Regional heat map of healthcare provider locations for all 192 patients based on the NPI Registry Practice by 5-digit zip code**


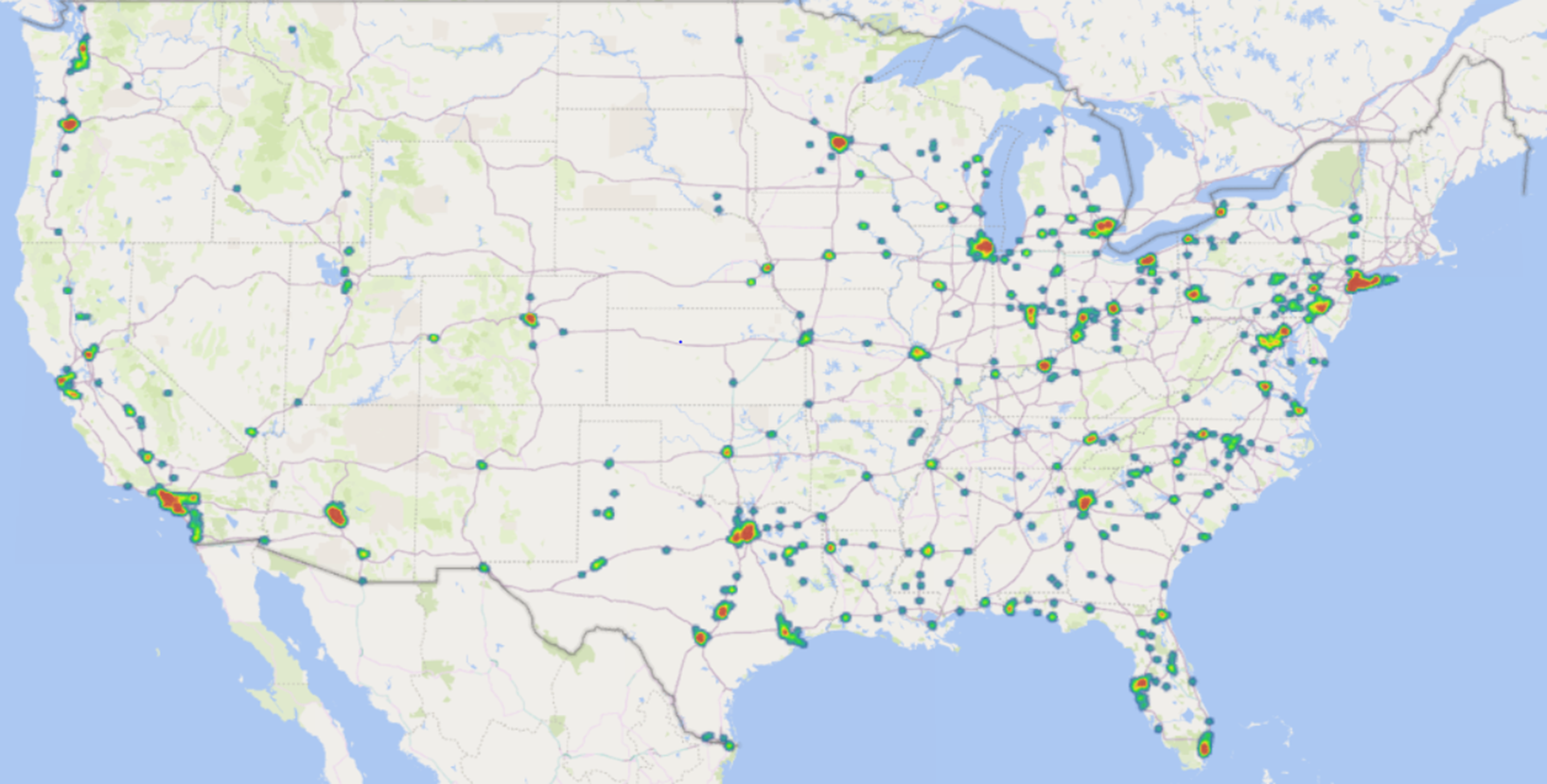


Table S1. Codes for planned vs unplanned hospitalization
Rules, with normal birth deliveries removed from consideration:

Unplanned hospitalization = any occurrence of one of the below codes.

Planned hospitalization = all other inpatient events.

| **Code** | **Description** | **Type** |
| --- | --- | --- |
| 31500 | Intubation, endotracheal, emergency procedure | CPT |
| 31603 | Tracheostomy, emergency procedure; transtracheal | CPT |
| 31605 | Tracheostomy, emergency procedure; cricothyroid membrane | CPT |
| 99281, 99282, 99283, 99284, 99285, 99288, 4084F | * | CPT |
| G0380, G0381, G0382, G0383, G0384 | * | HCPCS |
| G9752 | Emergency surgery | HCPCS |
| R40.2112 | Coma scale, eyes open, never, at arrival to emergency department | ICD10 |
| R40.2122 | Coma scale, eyes open, to pain, at arrival to emergency department | ICD10 |
| R40.2132 | Coma scale, eyes open, to sound, at arrival to emergency department | ICD10 |
| R40.2142 | Coma scale, eyes open, spontaneous, at arrival to emergency department | ICD10 |
| R40.2212 | Coma scale, best verbal response, none, at arrival to emergency department | ICD10 |
| R40.2222 | Coma scale, best verbal response, incomprehensible words, at arrival to emergency department | ICD10 |
| R40.2232 | Coma scale, best verbal response, inappropriate words, at arrival to emergency department | ICD10 |
| R40.2242 | Coma scale, best verbal response, confused conversation, at arrival to emergency department | ICD10 |
| R40.2252 | Coma scale, best verbal response, oriented, at arrival to emergency department | ICD10 |
| R40.2312 | Coma scale, best motor response, none, at arrival to emergency department | ICD10 |
| R40.2322 | Coma scale, best motor response, extension, at arrival to emergency department | ICD10 |
| R40.2332 | Coma scale, best motor response, abnormal, at arrival to emergency department | ICD10 |
| R40.2342 | Coma scale, best motor response, flexion withdrawal, at arrival to emergency department | ICD10 |
| R40.2352 | Coma scale, best motor response, localizes pain, at arrival to emergency department | ICD10 |
| R40.2362 | Coma scale, best motor response, obeys commands, at arrival to emergency department | ICD10 |
| R40.2442 | * | ICD10 |
| *Conditions, procedures, and hospitalizations are composed of 777 unique ICD-9/10 codes, 888 CPT/HCPCS and ICD-10 procedure codes, aggregated into appropriate groupings. | | |
